# Sharpness recognition based on synergy between bio-inspired nociceptors and tactile mechanoreceptors

**DOI:** 10.1038/s41598-021-81199-3

**Published:** 2021-01-22

**Authors:** Adel Parvizi-Fard, Nima Salimi-Nezhad, Mahmood Amiri, Egidio Falotico, Cecilia Laschi

**Affiliations:** 1 Medical Biology Research Center, Institute of Health Technology, Kermanshah University of Medical Sciences, Kermanshah, Iran; 2 Medical Technology Research Center, Institute of Health Technology, Kermanshah University of Medical Sciences, Parastar Ave., Kermanshah, Iran; 3 The BioRobotics Institute, Scuola Superiore Sant’Anna, Pontedera, Italy; 4 Department of Excellence in Robotics and AI, Scuola Superiore Sant’Anna, Pisa, Italy; 5 Department of Mechanical Engineering, National University of Singapore, Singapore, Singapore

**Keywords:** Biomedical engineering, Mathematics and computing, Nanoscience and technology

## Abstract

Touch and pain sensations are complementary aspects of daily life that convey crucial information about the environment while also providing protection to our body. Technological advancements in prosthesis design and control mechanisms assist amputees to regain lost function but often they have no meaningful tactile feedback or perception. In the present study, we propose a bio-inspired tactile system with a population of 23 digital afferents: 12 RA-I, 6 SA-I, and 5 nociceptors. Indeed, the functional concept of the nociceptor is implemented on the FPGA for the first time. One of the main features of biological tactile afferents is that their distal axon branches in the skin, creating complex receptive fields. Given these physiological observations, the bio-inspired afferents are randomly connected to the several neighboring mechanoreceptors with different weights to form their own receptive field. To test the performance of the proposed neuromorphic chip in sharpness detection, a robotic system with three-degree of freedom equipped with the tactile sensor indents the 3D-printed objects. Spike responses of the biomimetic afferents are then collected for analysis by rate and temporal coding algorithms. In this way, the impact of the innervation mechanism and collaboration of afferents and nociceptors on sharpness recognition are investigated. Our findings suggest that the synergy between sensory afferents and nociceptors conveys more information about tactile stimuli which in turn leads to the robustness of the proposed neuromorphic system against damage to the taxels or afferents. Moreover, it is illustrated that spiking activity of the biomimetic nociceptors is amplified as the sharpness increases which can be considered as a feedback mechanism for prosthesis protection. This neuromorphic approach advances the development of prosthesis to include the sensory feedback and to distinguish innocuous (non-painful) and noxious (painful) stimuli.

## Introduction

One of the main functions of the somatosensory system is to respond to the various types of tactile stimuli^1^. Touch sense provides valuable and essential contact information and allows us to interact with the environment and perform daily tasks^2^. Meissner corpuscles, Merkel cells, Ruffini endings, and Pacinian corpuscles are the primary skin mechanoreceptors that transmit tactile information to the upper layers of the nervous system. The Merkel cells and Ruffini endings are labeled as slowly adapting (SA) and respond to the sustained tactile stimuli. Meissner and Pacinian corpuscles which are known as rapidly adapting (RA) mechanoreceptors, respond to the onset and offset of the tactile stimulation^1,3^. These mechanoreceptors are innervated by the first-order neurons of the tactile pathway. The innervation pattern enables individual afferents to encode a portion of the geometric characteristics of the touched objects^4^. More recently, it is demonstrated that tactile information coding^5^ and tactile features extraction^4^ are also done by fingertip. Indeed, activation of tactile afferents spatially encodes the contact stimuli and sends the tactile information to the upper layers of the somatosensory pathway.

Free nerve endings (nociceptors) are placed in the exterior layer of the skin (epidermal layer) and are widely distributed over the body. They convey the tactile stimuli to the spinal cord leading to the perception of a painful experience^6^. Free nerve endings innervate the skin, bones, muscles, heart, and most of the internal organs. Nociceptors behave as high-threshold mechanoreceptors (HTMR) and respond to harmful stimuli through Aβ, Aδ, and C nerve fibers^3^. The mechanism of pain perception has individual peripheral receptors and includes a complex and chemically unique set of central circuits^7^. It has been demonstrated that pain perception is increased when nociceptors are active^7^. In this way, we can perceive a range of innocuous and noxious feelings.

Despite substantial progress in the design and control of prosthesis^8^, sensory perception of prosthetic hands is at the beginning of the road. Due to the importance of the tactile sense and its significant role in prostheses, it has undoubtedly attracted much attention to the development of new tactile sensors and bringing back sensory information in amputees. Recent studies focus on replicating the behavior of biological tactile receptors using sophisticated skin dynamics^9^ and neuromorphic systems^10^ to improve the efficiency and performance over traditional techniques. The flexible electronic elements^11–13^, self-healing^14,15^ recyclable materials^16^, mechanoreceptor-inspired elements^14,17^, and optoelectronic strain sensors^18^ have been proposed for prosthetic limbs. In this research, a novel neuromorphic system is designed and then tested by taking into account the biological features of mechanoreceptors and nociceptors for interpretation of tactile information.

Neuromorphic systems replicate the biological functions and spike-based neuronal processing and are broadly based on the analog and digital realization^19^. Neuromorphic sensory systems have made a great step forward in recent years using a new form of asynchronous output representation which provides timing information similar to the action potentials in the biological neuronal systems^20^. In the last few years, the application of spiking neural networks and neuromorphic implementations in tactile systems has been increased^10,21–23^. One of the most effective methods of realizing these computational neural models is digital circuit implementation due to their high performance for practical applications^24–30^. Digital execution with Field-Programmable Gate Array (FPGA) offers parallel computations and flexibility for algorithm investigation while filling time and performance limitations. FPGAs have broad applications in the neural network simulations^31^ and motivate further exploration^32,33^. An approximate circuit technique was used to implement tactile data processing on FPGA for the e-skin applications^34^. Furthermore, the spiking neural network implemented on FPGA was proposed for bi-directional interaction with living neurons cultured in microelectrode array^35^. The spiking model of cutaneous mechanoreceptor is implemented on the digital hardware (FPGA) to identify the distinct pressure stimuli^36^. For simulation and digital execution of the SA-I and RA-I afferents on the FPGA, the Izhikevich neuron model was frequently used in recent studies due to its rich dynamics which is suitable for tactile sense modeling^36,37^. Salimi-Nezhad and his colleagues^38^ implemented a population of afferents on the FPGA to realize the spatial coding and used a glove covered by pressure sensors to recognize objects during grasping. A neuromorphic system for pain perception and self-protection of a hand prosthesis was introduced by Osborn and his colleagues^37^. They fabricated a multilayered e-skin which imitates the behavioral characteristics of mechanoreceptors and nociceptors to provide sensory feedback for a prosthesis.

Given the fact that the majority of tactile information collected from the environment is encoded not only in multiple sub-modalities but also through a population of different afferent types, in the present research a bio-inspired digital system for the first layer of tactile sensory pathways including SA-I/RA-I afferents and nociceptors is designed. Specifically, the concept of the nociceptor is functionally implemented on the FPGA for the first time. One of the main features of tactile afferents is that their distal axon branches in the skin, creating complex receptive fields^39^. Consequently, the innervation concept to form receptive fields is also integrated into the proposed tactile neuromorphic systems. The digital afferents have receptive fields that overlap each other. To have a bio-inspired model for the SA-I/RA-I afferents and nociceptor, the Izhikevich neuron model is considered. Moreover, similar to the biological afferents which are not synaptically connected and only convey tactile information from the fingertip to the spinal cord for further processing, here, we have implemented a population of afferent circuits while considering the innervation concept to build receptive fields. Next, we investigate how the collaboration of afferents and nociceptors facilities sharpness recognition. It should be pointed out that utilizing the innervation technique in the prosthetic/robotic applications not only reduces the number of implemented afferents which in turn decreases the cost and power consumption of the neuromorphic devices but also high-resolution tactile sensors can also be handled. Furthermore, by implementing the nociceptors in the proposed tactile neuromorphic system, the concept of pain feeling also emerges. This can provide the prosthesis self-protection to avoid injury during haptic exploration. Indeed, sensor arrays are exposed to damage that can adversely affect the performance of the neuromorphic system^40^. Considering the role of nociceptors and mechanoreceptors, simultaneously, makes the system to be robust against damage in taxels or afferents/nociceptors to some extent. The bio-inspired tactile system includes a population of 23 digital afferents (12 RA-I, 6 SA-I, and 5 nociceptors). Using the proposed system, we explore how the collected spike responses can be used for sharpness classification. In particular, first, the impact of the afferent innervation and creation of receptive field on the firing pattern is investigated. Second, the contribution of tactile afferents and nociceptors on sharpness recognition is explored. Third, the fault tolerance characteristic of the biomimetic system is addressed.

## Procedure

**Figure 1:**
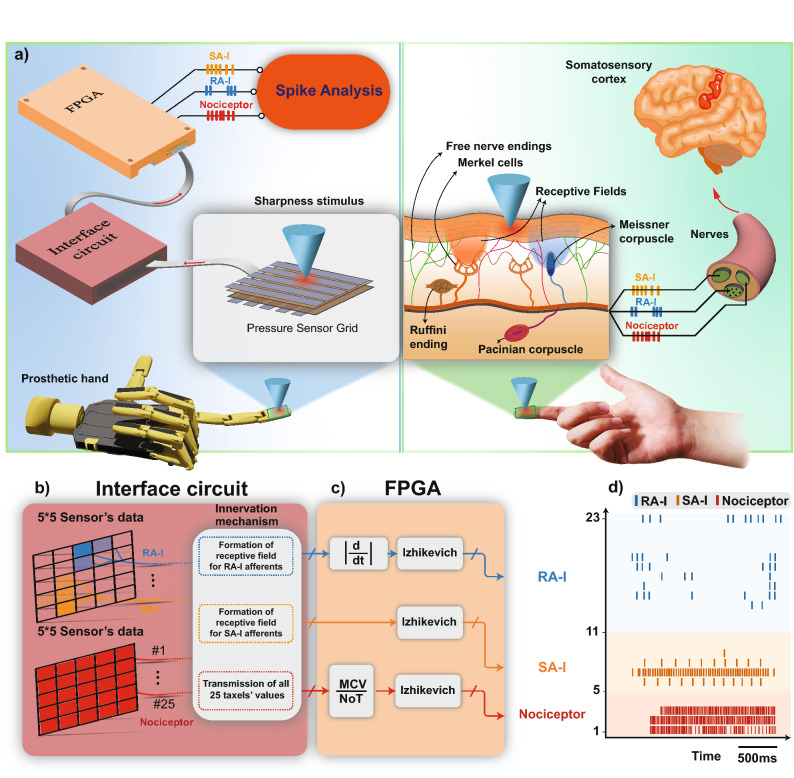
The proposed architecture for the FPGA-based tactile neuromorphic system. (**a**)-Left: Artificial pathway on the prosthetic hand that transduces the contact events to spiking responses in the parallel computational mechanism. The pressure signal obtained from the tactile sensor is converted to the current, I, (in the interface circuit) to feed as the input to the Izhikevich digital circuit (implemented on the FPGA). (**a**)-Right: Different mechanoreceptors distributed across the patch of skin. The afferent fibers transmit the tactile information obtained by the skin mechanoreceptors to the spinal cord. (**b**) The interface circuit connects the sensor array to the FPGA and performs innervation and receptive field creation for the digital afferents which are implemented on the FPGA. (**c**) The bio-inspired circuits of 12 RA-I, 6 SA-I, and 5 nociceptors are executed on the FPGA to generate the spike responses. The designed digital circuits convert the data from the interface circuit to the appropriate spike trains and send them to the PC by UART protocol. (**d**) The recorded firing patterns of the population of 23 biomimetic circuits from the FPGA for a sample of sharp objects.

The human tactile system converts the contact events at the fingertip to trains of action potentials (spikes) and then transmits to upper processing layers (Fig. 1a right). The biological SA-I afferents produce a sustained response to a static indentation of the skin^41^ and the biological RA-I afferents respond only to the onset and offset phases of indentation^42^. Similarly, we have developed a new communication architecture for e-skins that can functionally mimic the behavior of mechanoreceptors and afferents/nociceptors (Fig. 1a left). Tactile information is collected from the pressure sensor grid and then transmitted to the neuromorphic system through the interface circuit (Fig. 1b). A population of 23 afferents (12 RA-I, 6 SA-I, and 5 nociceptors) is digitally realized in the FPGA (Fig. 1c). The ratio of these two afferents is according to previous research^42^ and can be scaled up easily based on the applications. The data which are delivered to the FPGA comes from three groups. Individual digital SA-I afferent receives its inputs directly from the specified receptive field. For each digital RA-I afferent, from its receptive field, the derivative of the input signal is first calculated and then is rectified to be applied to the Izhikevich neuron model^22,38,43,44^. This is due to the fact that based on the biological evidence, the RA-I afferents respond to dynamic skin deformations, hence, for the trapezoidal indentation profile, the RA-I afferents are activated during the onset and offset phases. Individual digital nociceptor also receives the sensor data from all taxels. In this case, we detect the number of taxels (‘*NoT*’) that exceeds the predefined thresholds. Next, the maximum current value (‘*MCV*’) of the 25 taxels is determined and then the division of ‘*MCV*’ over ‘*NoT*’ is calculated (shift to the right in the FPGA). Finally, this value is applied to the Izhikevich neuron model to produce spikes. To analyze the tactile data, all 23 obtained spike trains are transmitted to the Personal Computer (PC) through the Universal Asynchronous Receiver-Transmitter (UART) interface. The spike trains of the digital afferents and nociceptors are illustrated in Fig. 1d and Movie S1.

**Figure 2:**
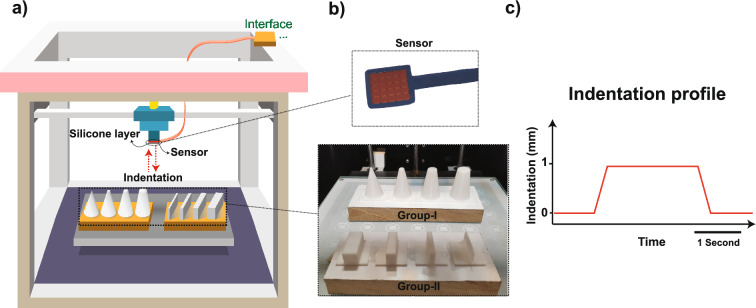
Illustration of the experimental setup which includes a robotic system with three-degree of freedom, tactile sensor, and 3D-printed objects. (**a**) The custom-built robot is equipped with a 5 × 5 tactile sensor. Red dash lines illustrate the touch protocol, where the sensor touches different objects. (**b**) Tactile sensor and 3D-printed objects. (**c**) Trapezoidal indentation profile. The hold phase of indentation lasts two seconds. Each object is touched by the tactile sensor 10 times and spike responses of the bio-inspired afferents/nociceptors are collected for further analysis.

To show the performance of the proposed bio-inspired tactile system in a real application, an experimental setup is developed. A 5 × 5 pressure tactile sensor is mounted on a custom-made robotic system with three-degree of freedom to touch the 3D-printed objects with different sharpness (Fig. 2a). Two groups of objects are employed in the experiments. Group-I is contained cone-shape objects with various sharpness and Group-II is included cube-shape objects with different widths (Fig. 2b) (objects are described in detail in “Methods”). The indentation track followed a trapezoidal profile and lasted 2500 ms in total, including 250 ms for onset, 250 ms for offset with constant speed, and 2000 ms for hold phase (Fig. 2c). An individual object has been touched by tactile sensor 10 times, and considering 8 objects (both Group-I and Group-II), 80 trials in total were recorded for the purpose of sharpness classification.

## Results

### Receptive fields of SA-I and RA-I afferents

**Figure 3:**
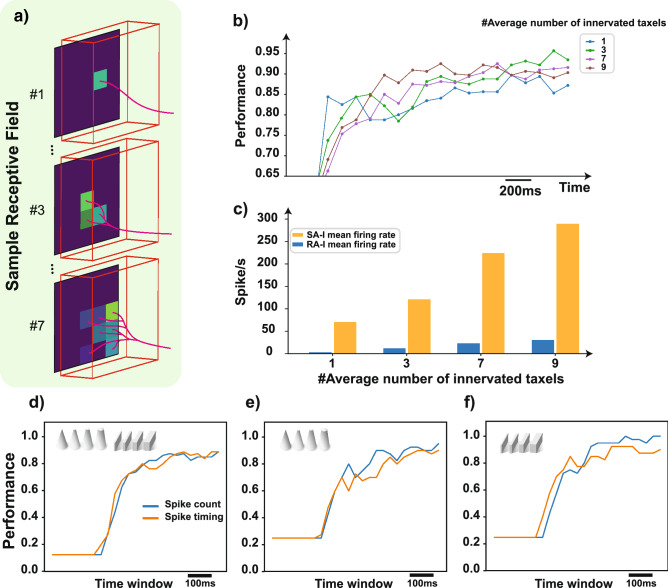
The impact of the innervation on the classification performance of the robotic data. (**a**) The samples of the receptive field for SA-I and RA-I afferents innervate the tactile sensor elements (taxels). (**b**) Classification performance of digital SA-I and RA-I afferents in distinct time windows when the receptive field size is changed. Spike count algorithm is used right after the sharp objects are touched by the sensor. (**c**) The firing rate with respect to the variation in receptive field size. Classification performance of (**d**) all objects, (**e**) Group-I objects, and (**f**) Group-II objects for the total population in different time windows using spike count (blue) and spike timing (orange) algorithms when three taxels are innervated by the digital afferents on average.

The 18 biomimetic digital circuits (6 SA-I and 12 RA-I) which have been implemented on the FPGA innervate the 25 channels of the tactile sensor through the interface circuit. In this case, spatially nearby taxels of the tactile sensor are connected to one afferent with different weights, creating complex receptive fields (Fig. 3a). Based on the experimental observation, the first-order neurons in the tactile sensory pathway branch in the skin and form many transduction sites^4^. This arrangement constitutes a peripheral neural system for signaling geometric features of the touched objects. Relying on this concept, we offer the innervation concept in the robotic applications between the sensor’s taxels and digital afferents. In other words, a few numbers of afferents are acquired to cover the whole area of the tactile sensor. The implemented digital afferents randomly innervate the mechanoreceptor grid (sensor’s elements) with different weights. The receptive fields also have overlaps which in turn produce diverse spiking responses. Now it is investigated how the afferents encode the stimuli into spiking activity utilizing the innervation mechanism. Some samples of receptive fields with various innervation patterns have been illustrated in Fig. 3a. For examining the afferents responses, the first 3-principal components of the feature space obtained from the spike count algorithm are extracted by Principal Component Analysis (PCA) as the inputs of the K-nearest neighbor (KNN) classifier (Fig. S1). Figure 3b shows the sharpness classification accuracy of 8 objects (Group-I and -II) when different numbers of tactile sensor elements including 1, 3, 7, and 9 taxels are innervated on average by one afferent in separate experiments. As can be seen, the innervation method improves the classification performance. By expanding the receptive field, the spiking activity of afferents is also increased (Fig. 3c). Increasing the number of innervated taxels decreases the unused taxels and thus the whole sensor area is covered by the afferents. It should be pointed out that digital afferents have randomly innervated the sensor taxels. Next, we analyze the obtained spike responses from a neuroscience point of view and consider two main protocols for neural processing: rate coding and temporal coding. For the former, the number of spikes (spike count) is computed and for the latter, using the spike temporal pattern we calculate the Victor and Purpura distance (spike timing). In this way, the objects are classified based on the firing patterns of the digital afferents/nociceptors at the population-level^45^ (“Methods”). Interestingly, the performance curves of both firing rate and spike timing algorithms, are similar and begin to increase after emission of the first few spikes (Fig. 3d–f). The results shown in Fig. 3d–f are based on the innervation of three taxels by each afferent. As can be seen, spatial coding is relatively stronger than the time coding for sharpness detection. Generally, these findings motivate the researchers to use the receptive fields in tactile neuromorphic devices in order to reduce the number of implemented artificial afferents when using high-resolution tactile sensors and consequently decrease the cost and power consumption.

### Contribution of tactile afferents and nociceptors

**Figure 4:**
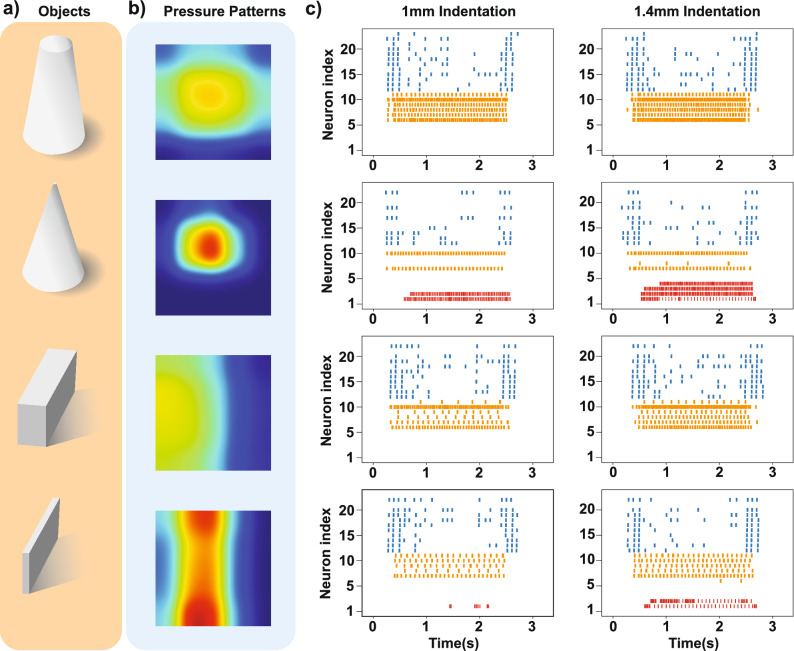
Responses of the bio-inspired SA-I/RA-I/nociceptors circuits to different objects. (**a**) Two different groups of objects with two different levels of sharpness are used to elicit responses. (**b**) Pressure pattern of the tactile sensor during indentation of each object by the custom-built robotic system. (**c**) Spiking activity of the bio-inspired afferents (RA-I (blue), SA-I (orange)) and bio-inspired nociceptor (red) for two levels of indentation (1 and 1.4 mm). As the object sharpness is increased, the spiking responses of the digital circuits of the SA-I/RA-I are decreased while firing patterns of the bio-inspired nociceptors are increased.

**Figure 5:**
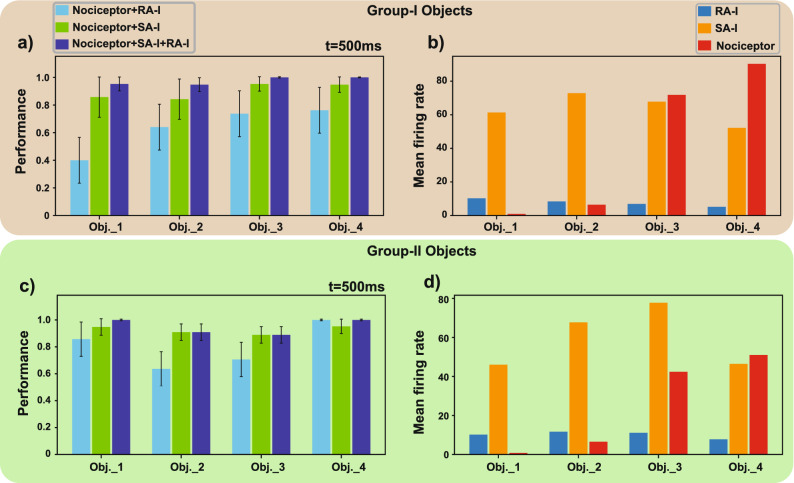
Contribution of the individual bio-inspired afferents and nociceptors in sharpness detection. Classification accuracy for each population of SA-I/RA-I/nociceptor and their combination for (**a**) Group-I objects and (**c**) Group-II objects. The mean firing rate of digital afferents and digital nociceptors during the response to (**b**) Group-I and (**d**) Group-II objects. For the sharpness classification, the spike count algorithm for 500 ms has been used.

Touching an object at low and moderate levels of physical contact is informative and can also be pleasant, however, it may be turned into noxious touch at a higher intensity. Pain perception helps us to avoid such situations. Nociceptors cause painful feelings to warn us that the body tissue is damaged or is in danger of being damaged. We demonstrate how the bio-inspired nociceptor model response to sharp object and also we assess the contribution of afferents and nociceptors in identifying the painful touch experience to protect the prosthetic hand from being damaged. The Izhikevich neuron model is used to mimic the spiking neural activity of tactile receptors^46^. It preserves neural dynamics while maintaining computational efficiency. In the Izhikevich neuron model, diverse firing patterns can be easily obtained by adjusting parameters and thus offers it as a good candidate for converting the obtained signals from sensor taxels to spike trains. It also has been used for the implementation of SA-I and RA-I afferents on the FPGA in recent researches^36,38^. To model the spiking responses of the SA-I/RA-I afferents and nociceptors, here, we use the regular spiking and fast spiking modes of the Izhikevich model, respectively. The dynamics are chosen to functionally adapt to the biological behavior of the SA-I/RA-I and free nerve endings^37^. When an object is touched by the tactile sensor array, a higher number of active taxels indicate a larger distribution of the pressure on the artificial fingertip, which is considered as an innocuous (non-painful) tactile stimuli. On the other hand, activating a lower number of taxels with high pressure is assumed as a noxious touch. Two cones and two cubes from Group-I and -II are shown in Fig. 4a. The activation patterns of the tactile sensor are illustrated in Fig. 4b when the objects are touched. The bio-inspired afferents/nociceptors which have been implemented on the FPGA encode the tactile stimuli in the spatiotemporal pattern of spiking activity. Figure 4c shows the spike responses recorded from the FPGA for two different indention profiles. Generally, deeper indention (stronger contact) leads to an increase in the firing rate. As can be seen in Fig. 4c, the nociceptors (neuron #1-5 shown in red) strongly respond to the sharper objects. Next, we investigate the contribution of each afferent/nociceptor, in sharpness detection. The results are illustrated in Fig. 5 for the spike count algorithm. The synergy between digital nociceptors and digital SA-I afferents shows better sharpness recognition compared to the collaboration between digital nociceptors and digital RA-I afferents (Fig. 5a,c). This may be caused by the inherent characteristics of the RA-I afferents which respond to the transient phase of contact (onset and offset of contact). In our experiment, the object indents into the sensor and remains unchanged until the end of the trial, hence, the RA-I afferents are silent during the holding phase (Fig. 4c). On the other hand, SA-I afferents provide consistent responses during the onset, hold phase, and offset of each trial (Fig. 4c). The mean firing rate of each bio-inspired SA-I/RA-I/nociceptor circuit is depicted in Fig. 5b,d. Interestingly, it shows that the sharper objects decrease the firing activity of bio-inspired SA-I/RA-I, while, increase the firing responses of the bio-inspired nociceptors. This is because of the involving fewer taxels for sharper objects.

### Fault-tolerance feature of the tactile neuromorphic system

**Figure 6:**
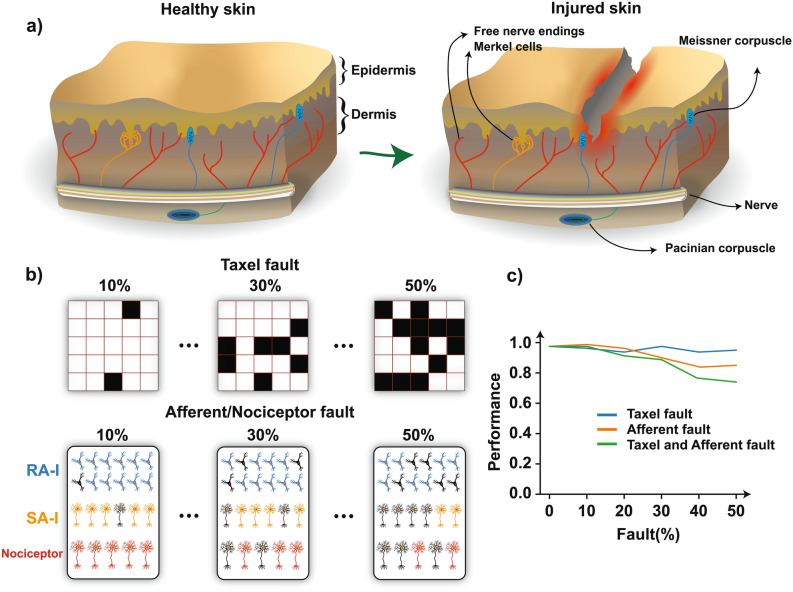
Robustness of the proposed bio-inspired system against taxel and afferent/nociceptor damage. (**a**) Schematic of the healthy and injured skin. (**b**) several taxels (top) and afferents (bottom) are randomly damaged (black squares and dark neurons, respectively). According to the fault level, the output signal of the damaged taxels is set to zero in the interface circuit (taxel fault experiment) or the digital neurons in the FPGA are deactivated and thus they cannot fire anymore (afferent/nociceptor fault experiment). (**c**) Classification performance for damaged taxels (blue), damaged neurons (orange), and both (green).

This section aims to explore the robustness of the proposed bio-inspired system. Figure 6a illustrates healthy and injured skin. Because of the skin injury, some of the mechanoreceptors are probably affected, however, we are still able to recognize different objects with the help of unaffected mechanoreceptors which show the robustness of the biological tactile system^47^. Fault-tolerance capability is an increasingly important feature for robotic/prosthetic hands, especially for remote applications^40,48,49^. Robustness in neuromorphic systems leads them to be more employed in a variety of applications. To examine the fault-tolerance characteristic of the proposed bio-inspired tactile system we perform two experiments. In the first one, we randomly select several taxels and making them defective and in the second experiment we randomly deactivate digital neurons in the FPGA (Fig. 6b) and thus they cannot fire and generate spikes anymore. For the taxel damage experiment, according to the fault level, we set the output signal of the number of taxels to zero in the interface circuit and thus these taxels will be disabled or damaged. Furthermore, we consider both cases in which both taxels and afferents are disabled.

Figure 6c shows that increasing the number of disabled taxels/afferents leads to a decrease in the general performance of the KNN classifier as is expected. Nevertheless, it illustrates that the proposed digital bio-inspired system has a fault tolerance characteristic. In this case, when the amount of fault in both afferents and taxels is increased up to 50%, the performance level is only decreased around 30% and thus 70% of input stimuli are correctly classified. Noteworthy, the chance level is about 12.5% (Fig. 6c). Indeed, even when there are some disabled taxels/afferents, the obtained spike trains from the bio-inspired tactile system contain enough information, and this is one of the main advantages of the innervation mechanism which has been employed in this neuromorphic structure. Therefore, with this amount of large damage, the classification performance is five times higher than the chance level.

## Conclusion

The bio-inspired technology is trying to add the skin-like sensory ability to robotic/prosthetic hands to provide information of the hand position (proprioception) and grip forces^44^. Recently, there are good achievements in the realization of tactile prosthesis and robotic hands. However, the current systems should be advanced by finding appropriate methods from biological mechanisms and then transferring to real operation. This research offered an innovative approach for manufacturing sensory systems and opened a new window for analysis of digital afferents and nociceptors from a neuroscience point of view. This technical approach artificially replicated the firing responses of the SA-I/RA-I afferents as well as nociceptors to be employed in the bio-robotic and prosthetic applications. Applying the concept of innervation and receptive field in robotic/prosthetic applications reduces the cost of afferent implementation and consequently, high-resolution tactile sensors can be derived by fewer artificial afferents.

The recorded spike trains from bio-inspired afferents/nociceptors were reliable and contained enough information to be able to decode the input stimuli with high accuracy. Indeed, it was shown that the synergy between bio-inspired SA-I/RA-I afferents and nociceptors, yields an acceptable accuracy in sharpness recognition even when several taxels or afferents were damaged. According to the results, we observed that disabling the number of taxels causes the innocuous tactile acts as a noxious stimulation. This outcome is similar to the injured skin experiment in which a harmless touch may be perceived as an unpleasant sensation.

The proposed bio-inspired tactile system can be easily scaled up based on the required accuracy and application. It is also possible to use other neuron models such as Adaptive Exponential Integrate-and-Fire (AdExIF) instead of the Izhikevich neuron model which needs low hardware resources for digital implementation and can be addressed in future studies.

## Methods

The tactile sensor was connected to a custom-made robot with three-degree of freedom and touched different objects. To collect the data, a sensor array with 5 rows and 5 columns (25 channels in total) was used and each sensor element was represented by a variable resistor. Eight 3D-printed objects with different sharpness ordered in two groups (Group-I, -II) were utilized for the experiment (Fig. 2b). The indentation direction followed a trapezoidal profile and lasted 2500 ms in total (Fig. 2c). The sensor raw data was converted to digital values using a 10-bit analog-to-digital converter (ADC) in the interface circuit. The data was then sent to the personal computer, using the Python environment, through serial communication.

Receptive fields for SA-I and RA-I afferents were formed as 5 × 5 coefficient matrices in the interface circuit. Then each element of the coefficient matrices was multiplied one to one by the corresponding taxel data. After that, the summation of the obtained matrix was considered as the input current for the individual SA-I afferent. For the RA-I afferents, after getting the summation, the derivative and rectification operations were also applied and then fed as the input current to the Izhikevich digital circuit implemented on the FPGA. The entire data collected from the tactile sensor were transmitted to the digital nociceptors on the FPGA to produce spike responses functionally compatible with the biological observation.

To analyze the spiking activity of the afferents, we used spike count and spike timing methods. By applying PCA, the dimension of the feature space was reduced to three. As shown in Fig. S1, features are appropriately clustered in separate groups and the desired classes can be accurately recognized by conventional classifiers such as KNN. Figure S2 shows that there is no significant difference between the performance of the KNN and Support Vector Machine (SVM) classifiers, although the performance of the KNN classifier is a little bit better for longer time windows. For the KNN, K was set to five. For classification, 80% of samples were randomly grouped to serve as the training set and the remaining 20% samples were considered as the test set. Fivefold cross-validation was also used. The data samples were divided into 5 subsets. Each time, one of these 5 subsets was used as the validation set and the remaining 4 subsets formed the training set. Finally, the average performance across all 5 trials for each subset was computed.

### Tactile sensor

We used a custom-built tactile sensor consisting of a force-sensitive conductive material that was sandwiched between two layers of conductive traces as rows and columns. The tactile sensor has 5 rows and 5 columns arranged with 2.8 mm pitch (14 mm × 14 mm active area). The sensor was covered by a silicone layer to disperse the indentation force. As pressure was applied, the resistance decreased and then returned to its original value as the pressure was removed. In this work, the sensor grid played the role of both nociceptors and mechanoreceptors.

### Setup

The custom-built robotic setup was consisted of three stepper motors correspond to each axis (X, Y, Z). The movement resolution was about 10 µm in each direction. The sensor was placed on the Z-axis and moved vertically to touch the 3D-printed objects.

### Stimuli objects

Two groups of objects were used. The first group (Group-I) were four cone-shaped objects with a flat base diameter of 20 mm for all and the apex diameters of 1, 3, 5, and 9 mm. The second group (Group-II) were four cube-shaped objects with a length of 20 mm for all and the widths were 1, 3, 5, and 9 mm. These objects were fabricated using 3D printing technology.

### Interface circuits

The readout circuit was comprised of two analog multiplexers and a 32-bit ARM core microcontroller with a 10-bit ADC. Analog multiplexers were utilized to determine the rows and columns of the tactile sensor for data collection. The ADC converted the output of each taxel (sensor element) to the digital signals and the microcontroller transferred the data to the FPGA.

### Hardware implementation

The digital SA-I/RA-I afferents and nociceptors were implemented on the ZedBoard (a particular ZYNQ evaluation board). The ZedBoard is composed of two major sections: Programmable Logic (PL) and Processing System (PS). The PL section is a reconfigurable digital platform and the PS section is a dual-core ARM cortex-A9 processor. We used the PS section during data collection and data transferring to the personal computer using the UART interface. When the whole data was stored in the personal computer, then the classification was carried out offline. The purpose of the offline analysis is to show that obtained spike responses are informative and convey information in their spatiotemporal pattern.

Izhikevich neuron model is a compromise between the leaky integrate-and-fire (LIF) neuron model and Hodgkin-Huxley (HH) neuron model because of its efficient mathematical computations and diverse dynamical responses. In this research, the Izhikevich spiking model was first discretized using the Euler method, and then the digital circuit was implemented on FPGA.

The Izhikevich model is described as follows^46^:

1 $${v}^{{\prime}}=0.04{v}^{2}+5v+140-u+{k}_{s}\frac{I}{{C}_{m}}, \quad where \;\;\;(s=1, 2)$$

2 $${u}^{{\prime}}=a\left(bv-u\right)$$

3 $$if v\ge 30\;\;\text{mV} \to then \left\{\begin{array}{l}v \leftarrow c\\ u \leftarrow u+d\end{array}\right.$$

*v* and *u* are the membrane potential of the neuron and the membrane recovery variable, respectively. *I* is the input current. *a, b, c,* and *d* are the constant neuron parameters. ${k}_{s}$ scales the input current. For s = 1 the neuron has the regular spiking dynamic and when s = 2 the neuron is in the fast spiking mode. ${k}_{1}$ and ${k}_{2}$ are equal to 1/32 and 1/8, respectively. C_m_ is the capacitance value for dimensionality consistency and is equal to 1 F. The parameter values of the Izhikevich model for two dynamics including regular spiking and fast spiking were listed in Table 1. These parameters were adapted and taken from^46^.

**Table 1:** Parameter values of the Izhikevich neuron model.

Parameter	Regular spiking	Fast spiking
a	0.02 s^−1^	0.1 s^−1^
b	0.2 s^−1^	0.2 s^−1^
c	− 65 mV	− 65 mV
d	8 mV	2 mV

Equations (1)–(3) with regular spiking dynamic were used to describe the spiking part of the SA-I afferent model^36^ and with the fast spiking dynamic were used for nociceptors^37^.

The methodology proposed by Cassidy et al.^50^ is an efficient way to implement digital circuits with less hardware utilization. Following this method, we multiply Eq. (1) by 0.78125 which simplifies the parameter values to be with the power of two and hence facilitates the digital implementation. In this way, Eq. (1) can be rewritten as follows:

4 $${v}^{{\prime}}=\left(\frac{1}{32}\right){v}^{2}+4v+109.375-u+{k}_{s}\frac{I}{{C}_{m}}, \quad where \;\;\;(s=\text{1,2})$$

For the RA-I afferent, we utilize the method reported in^38^, and obtain the following equations for spike generation:

5 $${v}^{{\prime}}= \left(\frac{1}{32}\right){v}^{2}+4v+109.375-u+{k}_{3}\frac{\tau }{{C}_{m}}I{^{\prime}}$$

6 $${u}^{{\prime}}=a\left(bv-u\right)$$

7 $$if v\ge 30\;\;\text{mV} \to then \left\{\begin{array}{l}v \leftarrow c\\ u \leftarrow u+d\end{array}\right.$$

${k}_{3}$ is a constant factor that scales the input and τ is the time constant and their values are 128 and 1 s, respectively. It should be pointed out that the regular spiking dynamic is also used for the RA-I afferent model.

To implement the digital circuits, the Euler method was utilized to discretize the differential equations. The discretizing step was dt = 1 ms. Discretizing the Eqs. (2)–(4) for the SA-I afferent and nociceptor yields:

8 $$v\left[n+1\right]=v\left[n\right]+\left(\left(\frac{1}{32}\right)*v\left[n\right]*v\left[n\right]+4*v\left[n\right]+109.375-u\left[n\right]+{k}_{s}*I\left[n\right]\right), \quad where\;\; (s=\text{1,2})$$

9 $$u\left[n+1\right]=u\left[n\right]+a*\left(b*v[n]-u[n]\right)$$

10 $$if v\left[n+1\right]\ge 30\;\; \text{mV} \to then \left\{\begin{array}{l}v[n+1] \leftarrow c\\ u[n+1] \leftarrow u[n]+d\end{array}\right.$$

Similarly, for the RA-I afferent, Eqs. (5)–(7) are discretized as follows:

11 $$v\left[n+1\right]=v\left[n\right]+ \left ( \left(\frac{1}{32}\right)*v\left[n\right]*v\left[n\right]+4*v\left[n\right]+109.375-u\left[n\right]\right)+{k}_{3}*(I\left[n+1\right]-I[n])$$

12 $$u\left[n+1\right]=u\left[n\right]+a*\left(b*v[n]-u[n]\right)$$

13 $$if v[n+1]\ge 30\;\text{mV} \to then \left\{\begin{array}{l}v[n+1] \leftarrow c\\ u[n+1] \leftarrow u[n]+d\end{array}\right.$$

The register length was N = 32 on the FPGA (1 bit for sign, 13 bits for the integer part, and 18 bits for the fractional part) to obtain an acceptable trade-off between hardware utilization and precision^36^. Hardware utilization for the neuromorphic system is presented in Table 2.

**Table 2:** Hardware utilization for the neuromorphic system.

	Used	Available
Slice LUTs	34,391 (64%)	53,200
Slice registers	4542 (4%)	106,400
Slice	12,191(91%)	13,300
LUT as logic	34,329(64%)	53,200
LUT as memory	62(1%)	17,400
LUT flip flop pairs	1331 (2%)	53,200
DSP48E1	69 (31%)	220
Bonded IO	17 (8%)	200

### Spike timing analysis in population-level

For decoding based on temporal information of the spike patterns, the Victor and Purpura distance (VPd)^45^ was used. This metric is a measure of spike-train synchrony by computing the minimal cost necessary to transform one spike train into another, employing basic operations (spike deletion, spike insertion, spike shift). A detailed description of the VPd method was reported in^51^.

## Supplementary Information

Supplementary Information.

Supplementary Video S1.

## Data Availability

All data are available from the corresponding author upon reasonable request.

## Code availability

All analyses reported in this work were made with custom code written in Python 3.7 and will be available from the corresponding author upon reasonable request. Moreover, the last version has been uploaded at: https://github.com/Research-lab-KUMS/Sharpness-Recognition.
