# Supplementary material for: Sharpness recognition based on synergy between bio-inspired nociceptors and tactile mechanoreceptors
Source: Sci Rep. 2021 Jan 22;11:2109. doi: 10.1038/s41598-021-81199-3 (PMC7822817; doi:10.1038/s41598-021-81199-3)
Supplement: Supplementary file 1 — Supplementary Information. [file 41598_2021_81199_MOESM1_ESM.docx]

**Sharpness Recognition Based on Synergy between**

**Bio-inspired Nociceptors and Tactile Mechanoreceptors**

Adel Parvizi-Fard^1^, Nima Salimi-Nezhad^1^, Mahmood Amiri^2*^, Egidio Falotico^3^ and Cecilia Laschi^3^

1 Medical Biology Research Center, Institute of Health Technology, Kermanshah University of Medical Sciences, Kermanshah, Iran

2 Medical Technology Research Center, Institute of Health Technology, Kermanshah University of Medical Sciences, Kermanshah, Iran

3 The BioRobotics Institute, Scuola Superiore Sant’Anna, Pontedera, Italy

* Corresponding author,

Email: [ma_amiri_bme@yahoo.com](mailto:ma_amiri_bme@yahoo.com)

**Additional information**


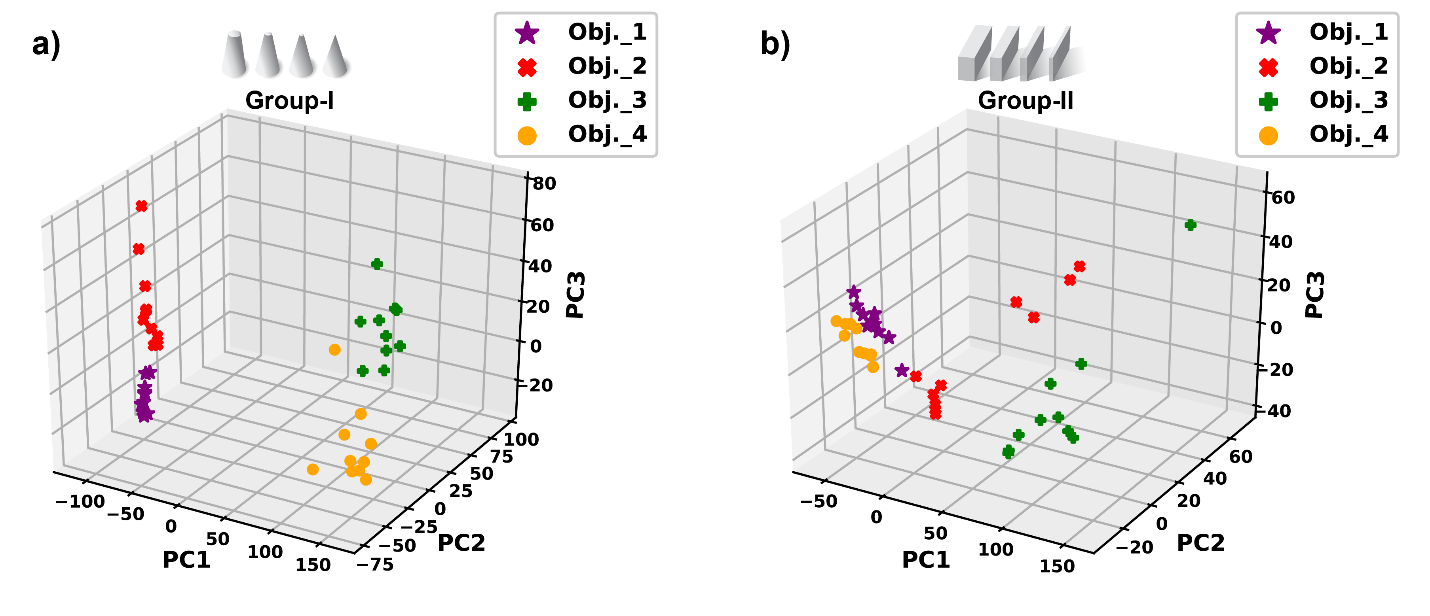


**Figure S1.** Principal component analysis. The first three principal components (PCs) were obtained from spike responses of (a) Group-I and (b) Group-II.


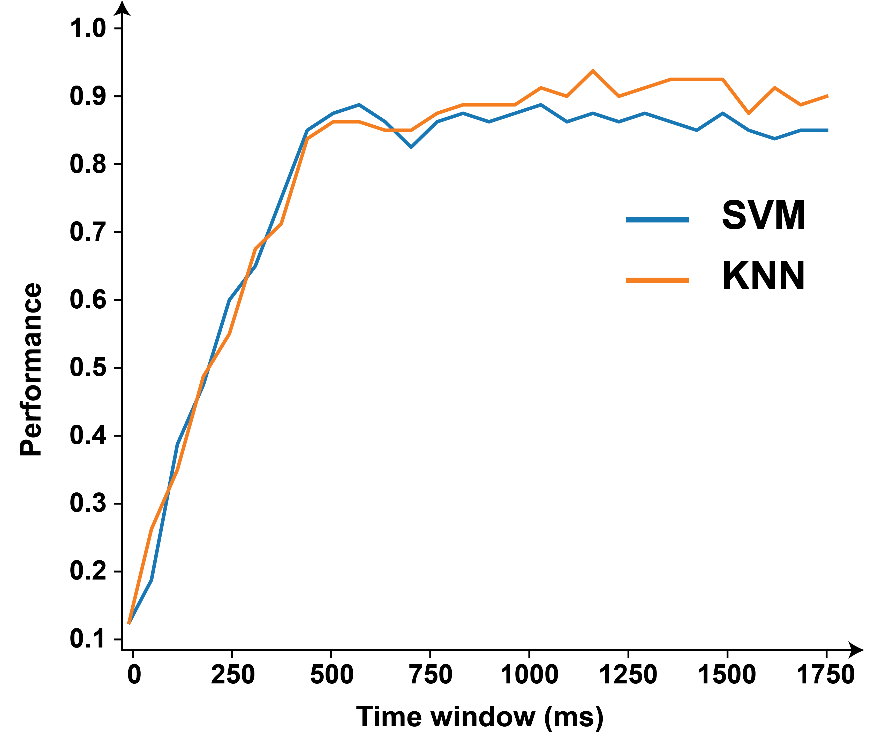


**Figure S2.** Classification performance of 8 objects (Group-I, -II) for different time windows right after the contact. The performance of SVM (blue) and KNN (orange) classifiers are almost similar with little improvement for KNN.

**
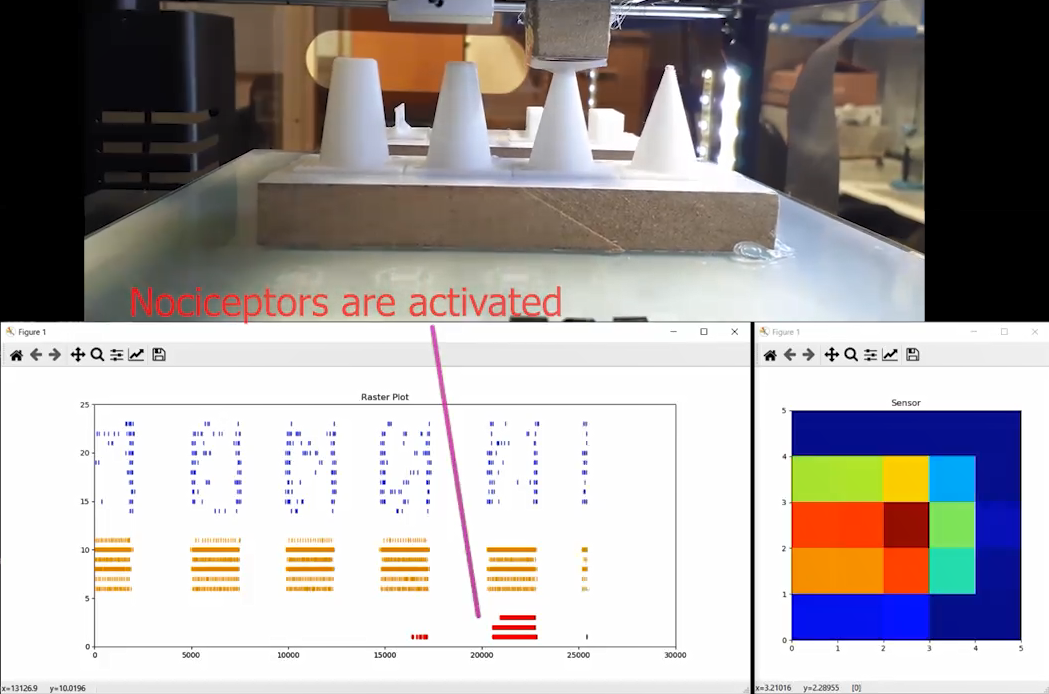
**

**Movie S1 –** The firing patterns of the bio-inspired digital circuits for the SA-I, RA-I, and nociceptors. The tactile sensor was installed on a robotic system and touched different 3D-printed objects.
